# Increased complement activation 3 to 6 h after trauma is a predictor of prolonged mechanical ventilation and multiple organ dysfunction syndrome: a prospective observational study

**DOI:** 10.1186/s10020-021-00286-3

**Published:** 2021-04-08

**Authors:** Ingrid Nygren Rognes, Søren Erik Pischke, William Ottestad, Jo Røislien, Jens Petter Berg, Christina Johnson, Torsten Eken, Tom Eirik Mollnes

**Affiliations:** 1 Department of Research, The Norwegian Air Ambulance Foundation, Oslo, Norway; 2 Institute of Clinical Medicine, Faculty of Medicine, University of Oslo, Oslo, Norway; 3 Department of Anaesthesiology, Division of Emergencies and Critical Care, Oslo University Hospital, Oslo, Norway; 4 Department of Immunology, Oslo University Hospital and University of Oslo, Oslo, Norway; 5 Faculty of Health Sciences, University of Stavanger, Stavanger, Norway; 6 Research Laboratory, Nordland Hospital, K.G. Jebsen TREC, Faculty of Health Sciences, The Arctic University of Norway, Bodø and Tromsø, Norway; 7 Centre of Molecular Inflammation Research, Norwegian University of Science and Technology, Trondheim, Norway

**Keywords:** Wounds and injuries, Humans, Complement activation, Complement membrane attack complex, Systemic inflammatory response syndrome, Multiple organ failure, Mortality

## Abstract

**Background:**

Complement activation is a central mechanism in systemic inflammation and remote organ dysfunction following major trauma. Data on temporal changes of complement activation early after injury is largely missing. We aimed to describe in detail the kinetics of complement activation in individual trauma patients from admission to 10 days after injury, and the association with trauma characteristics and outcome.

**Methods:**

In a prospective cohort of 136 trauma patients, plasma samples obtained with high time resolution (admission, 2, 4, 6, 8 h, and thereafter daily) were assessed for terminal complement complex (TCC). We studied individual TCC concentration curves and calculated a summary measure to obtain the accumulated TCC response 3 to 6 h after injury (TCC-AUC_3–6_). Correlation analyses and multivariable linear regression analyses were used to explore associations between individual patients’ admission TCC, TCC-AUC_3–6_, daily TCC during the intensive care unit stay, trauma characteristics, and predefined outcome measures.

**Results:**

TCC concentration curves showed great variability in temporal shapes between individuals. However, the highest values were generally seen within the first 6 h after injury, before they subsided and remained elevated throughout the intensive care unit stay. Both admission TCC and TCC-AUC_3–6_ correlated positively with New Injury Severity Score (Spearman’s rho, *p*-value 0.31, 0.0003 and 0.21, 0.02) and negatively with admission Base Excess (− 0.21, 0.02 and − 0.30, 0.001). Multivariable analyses confirmed that deranged physiology was an important predictor of complement activation. For patients without major head injury, admission TCC and TCC-AUC_3–6_ were negatively associated with ventilator-free days. TCC-AUC_3–6_ outperformed admission TCC as a predictor of Sequential Organ Failure Assessment score at day 0 and 4.

**Conclusions:**

Complement activation 3 to 6 h after injury was a better predictor of prolonged mechanical ventilation and multiple organ dysfunction syndrome than admission TCC. Our data suggest that the greatest surge of complement activation is found within the first 6 h after injury, and we argue that this time period should be in focus in the design of future experimental studies and clinical trials using complement inhibitors.

**Supplementary Information:**

The online version contains supplementary material available at 10.1186/s10020-021-00286-3.

## Background

Trauma is one of the main causes of premature death world-wide (GBD 2019 Diseases and Injuries Collaborators 2020). The innate immune response initiated by trauma, a response evolved to restore homeostasis after mild and moderate injuries, can be exaggerated and sustained in patients with severe trauma. Severely injured patients who survive the initial insult may suffer from detrimental systemic inflammation and subsequent remote organ dysfunction. Tissue injury leads to immediate release of endogenous danger signals that trigger a cascade of innate immune pathways, potentially leading to a systemic inflammatory response and multiple organ dysfunction syndrome (MODS) (Lord et al. 2014). MODS can be evident as early as at the day of admission in severely injured patients, and patients presenting with MODS have a prolonged intensive care unit (ICU) stay and increased mortality (Cole et al. 2020). The extent and immediacy of the immunological response is evidenced by genome-wide analyses of human blood leukocytes, showing an alteration in immune cell genes already within 2 h after injury (Cabrera et al. 2017).

The complement system is a crucial sensor of cellular stress and damage (Matzinger 2002; Hajishengallis et al. 2017; Huber-Lang et al. 2018), and its role in trauma has been investigated in human observational studies since the 1980 s (Fosse et al. 1987, 1998; Zilow et al. 1992; Donnelly et al. 1994; Meade et al. 1994; Roumen et al. 1995; Hecke et al. 1997; Ganter et al. 2007; Amara et al. 2009; Burk et al. 2012; Li et al. 2019; Huber-Lang et al. 2013; Chakraborty et al. 2018). Excessive complement activation has been reported within minutes after major trauma (Burk et al. 2012). A rapid consumption of complement factors and severe dysfunction of regulatory mechanisms can be seen in some severely injured patients, a state termed complementopathy (Amara et al. 2009; Burk et al. 2012).

Activation of complement occurs through three pathways; the classical, the lectin, and the alternative pathway, however, there is debate regarding which pathway is the most important in trauma (Ganter et al. 2007; Li et al. 2019). All three pathways converge at the central complement components (C) C3 and C5, with subsequent formation of the anaphylatoxins C3a and C5a and the terminal C5b-9 complement complex (TCC). TCC exists in two forms. The membrane-inserted form, named the membrane attack complex (MAC), can lyse bacteria and cells, but also activate the host’s own cells in sub-lytic doses (Morgan 2015), triggering further inflammation. The soluble form of TCC found in plasma (sC5b-9) is regarded biologically inert, but is the most robust activation product to evaluate systemic complement activation (Bergseth et al. 2013). It has an in vivo half-life of one hour, is relatively stable in vitro, and reflects the end stage of complement activation (Harboe et al. 2011).

Excessive complement activation after trauma has been associated with development of sepsis (Schoffel et al. 1991), acute respiratory distress syndrome (Zilow et al. 1992; Donnelly et al. 1994; Meade et al. 1994), systemic inflammation (Fosse et al. 1998) and MODS (Roumen et al. 1995; Ganter et al. 2007; Nuytinck et al. 1986), and is moreover associated with increased mortality (Roumen et al. 1995; Hecke et al. 1997; Ganter et al. 2007), increasing Injury Severity Score (ISS) and increasing physiological derangement (decreasing Base Excess, BE) (Fosse et al. 1998; Ganter et al. 2007; Sharma et al. 2004). Complement activity during the ICU stay has also been studied (Risberg et al. 1986; Zilow et al. 1992; Donnelly et al. 1994; Meade et al. 1994; Roumen et al. 1995; Hecke et al. 1997; Fosse et al. 1998; Amara et al. 2009; Burk et al. 2012; Li et al. 2019). However, most of these studies have only described and tested measurements of complement activation on group level at individual standardized time points, despite having a repeated-measures design. This approach poses a range of statistical problems (Matthews et al. 1990), and any information on individual concentration kinetics is lost. Further, the pathology after trauma is complex and heterogeneous with myriads of molecules released, and measurements at single time points are insufficient to describe relationships between time-varying molecular responses in full. Summary measures generated from repeated samples, e.g. area under individual concentration curves, have been proposed as useful alternatives in repeated measures analyses (Matthews et al. 1990).

There is an increasing interest in complement inhibition as a potential therapeutic strategy for trauma-induced systemic inflammation and MODS, but so far only a few experimental studies have been performed (Huber-Lang et al. 2018; Ricklin et al. 2018; Ricklin and Lambris 2013; Karasu et al. 2019). More knowledge on complement kinetics in human trauma is necessary to improve pathophysiological understanding, elucidate the clinical effects of complement activation and theoretically explore the potential benefit of complement inhibition, before interventional studies with complement inhibitors can be initiated (Karasu et al. 2019; Groeneveld et al. 2011).

The aim of this study was to investigate temporal changes of the complement activation end product TCC in high time-resolution samples from trauma patients in the first critical hours, followed by daily samples up to 10 days following injury, in order to describe its concentration kinetics and the association with anatomical injury, physiological derangement and outcome.

## Methods

### Study design and approval

This prospective observational study investigated concentration kinetics of the complement activation product TCC in serially collected plasma samples from 136 trauma patients. The study is part of a greater effort to describe the innate immune response following trauma, design and methods have been described in detail previously (Ottestad et al. 2019). Reference TCC values were obtained from 20 healthy volunteers. All parts of the study were approved by the Regional committee for medical and health research ethics (2010/2014 REK Sør-Øst D), in accordance with the Declaration of Helsinki. We adhered to Strengthening the Reporting of Observational Studies in Epidemiology (STROBE) statement for cohort studies (von Elm et al. 2007).

### Setting and participants

Patients were recruited by convenience at Oslo University Hospital (OUH) Ullevål, a Norwegian Level 1 trauma centre, from January 2011 through January 2014. All patients ≥ 18 years old who met criteria for trauma team activation were eligible for inclusion. Pregnant women and patients with burn injuries were not included. Patients were enrolled on arrival and followed through their ICU stay, limited to 10 days or death. Survival status at 30 days after injury was verified from the Norwegian Population Registry.

### Sample collection

Blood was drawn in K_2_EDTA-coated tubes after a discard tube, immediately after admission, 2, 4, 6 and 8 h thereafter, and every morning in the ICU for up to 10 days. Sampling through an arterial cannula was preferred in order to obtain blood that was not draining from any particular injured body part. All samples were handled as recommended for analyses of complement activation products (Mollnes et al. 1988). The EDTA tubes were put directly in ice slush after 8–10 inversions and centrifuged within 15 min at 2500 g for 15 min at 4 °C. The supernatant was immediately transferred to sterile polypropylene tubes and stored at – 80 °C. Peripheral venous samples from healthy volunteers were handled by the same protocol.

### Laboratory analyses

Complement activation was assessed by quantifying plasma TCC (sC5b-9) using an enzyme-linked immunosorbent assay (ELISA) as described originally (Mollnes et al. 1985) and later modified (Bergseth et al. 2013). The analyses were conducted blinded to the clinicians and the clinical data. In brief, TCC formation was quantified by use of the monoclonal antibody aE11 reacting with a neoepitope of C9 exposed when incorporated into fluid-phase sC5b-9 complex. Biotinylated anti-C6 mAb (clone 9C4) was used as detection antibody. Optimal sample dilution was determined to be 1:5. Values are given in arbitrary units (AU)/mL refereed to a standard curve of zymosan activated human serum defined to contain 1000 AU/mL. The standard curve and internal controls were based on the International Complement Standard #2 (Bergseth et al. 2013), made in our laboratory and stored in a biobank in Heidelberg, available to all complement laboratories worldwide. Intra and inter-assay coefficients of variation were 5 % and 10 %, respectively. In the present study the whole population was analysed in one batch, except for 12 patient samples analysed initially as a pilot.

In contrast to many other complement components, we have found that TCC does not change as function of sex and age. In the method paper (Bergseth et al. 2013) we investigated 10 males and 10 females from each of the age decades 20–70 (healthy registered blood donors) and found exactly the same reference range in all subpopulations, so these data could be pooled for a general normal reference range (Bergseth et al. 2013). The sample could be thawed and frozen 10 times without any change in the value (Bergseth et al. 2013). TCC is a particularly stable activation product, which can been stored for up to 10 years at − 70 °C. We collected a pool of healthy blood donors in 1985, thawed them and compared them in the same assay set up with a fresh pool of healthy blood donors, and the values were identical (unpublished observations). Furthermore, we have non-published observations from patients undergoing cardiopulmonary bypass with a substantial increase in TCC. Venous and arterial samples showed exactly the same values. It is reasonable to assume that this applies also for trauma patients despite being a different clinical situation as TCC is an inert molecule without receptors and circulates freely in plasma.

### Clinical data and routine laboratory analyses

Clinical data were collected from the Oslo University Hospital Trauma Registry, ICU charts and routine blood samples. All outcome measures and predictor variables were predefined (Osler et al. 1997; Balogh et al. 2000; Ouellet et al. 2012).

Primary outcome was ventilator-free days (VFD), defined as days alive and off ventilator during the first 30 days after trauma (Schoenfeld et al. 2002; Yehya et al. 2019). Patients transferred to another hospital while still intubated were regarded as ventilator treated through the rest of the 30-day period. Secondary outcome measure was daily Sequential Organ Failure Assessment (SOFA) score (Vincent et al. 1998) (Methods in Additional file 1). New Injury Severity Score (NISS) was chosen as a measure for overall injury severity (Osler et al. 1997; Balogh et al. 2000), and admission BE, assessed by arterial blood gas analysis within minutes of arrival in the trauma room (Cobas b 221 blood gas system, Roche Diagnostics Norway AS), as the primary measure for physiological derangement (Ouellet et al. 2012). Remaining predictor variables were admission TCC concentration and area under the individual TCC concentration curves during the time period from 3 to 6 h after injury (TCC-AUC_3–6_) (Ottestad et al. 2019), sex, age, and mechanism of injury (blunt or penetrating).

Patients with major head injury receive organ support due to the head injury per se, thereby affecting both VFD and SOFA score. Patients with and without an Abbreviated Injury Scale (AIS) severity code ≥ 3 in ISS region Head or neck were therefore analysed as separate populations.

### Statistical analyses

All sampling times were converted to elapsed time from injury. Concentrations of TCC were linearly interpolated to enable comparison of data at specific time points after injury across our asynchronously sampled data (Ottestad et al. 2019). The linearly interpolated dataset was also used to calculate a summary measure, area under the individual TCC curve 3–6 h after injury, and to assess Daily TCC. Details are given in Methods in Additional file 1.

Correlation between continuous variables was assessed by Spearman’s rank correlation coefficient (*ρ*). Group comparisons were performed by Fisher’s exact test for categorical data and Wilcoxon rank-sum test (Mann-Whitney) for continuous data.

Relationships between predictor and response variables were examined in multivariable linear regression models with backward elimination. The optimal model was selected using the Bayesian information criterion (BIC) (Neath and Cavanaugh 2012). Assessment of importance of the individual predictor variables was performed as variance-based sensitivity analysis (Saltelli 2002). Importance indices were constructed from observed combinations of factor values, since predictor variables were generally correlated. There were indications of non-linear associations between independent and dependent variables, and generalised additive models (GAMs) (Wood 2017), a generalisation of traditional linear regression models, were also explored, without significant impact on results.

A two-tailed *p* value ≤ 0.05 was chosen to represent statistical significance. Data analyses were undertaken using JMP 13.2.0 (SAS Institute, Cary, NC). GAM was performed using the function gam in the mgcv package version 1.8–17 in R 3.3.3 through the GAMs V1.0 JMP add-in.

### Extreme outlier TCC values

Three patients had extreme outlier TCC values, Fig. S1 in Additional file 2. One patient with minor injuries, NISS 5 and BE -2.6, had admission TCC concentration 14.1 AU/mL and a similarly elevated level two hours later. It was suspected that reliable values were not possible to obtain, conceivably due to heterophilic antibodies in the patient’s plasma (Bolstad et al. 2013). Two other patients had TCC levels above upper detection limit for the assay that could not be diluted to measurable levels, therefore invalidating calculation of area under the TCC concentration curves. The three patients are included in descriptive statistics but excluded from figures, comparisons, correlation and regression analyses.

## Results

### Study population

Out of 145 originally enrolled patients, 136 constituted the study population (Fig. S1 in Additional file 2), identical to the one reported in our previous paper (Ottestad et al. 2019). Demographics and clinical characteristics for the total population are given in Table 1, and the corresponding data for differences between patients with or without major head trauma in Table S1 in Additional file 3. Twenty patients died, 16 during the first 48 h, mainly due to major brain injury (*n* = 9) and excessive haemorrhage (*n* = 5) (Fig. S2 in Additional file 4).

**Table 1:** Characteristics of study population and controls

Characteristics	Trauma patients (*n =* 136)^a^	Healthy controls (*n =* 20)
Demographics		
Sex (male : female)	101 : 35	11 : 9
Age (years)	40 (18–94); *n =* 135	39 (22–58)
Preinjury ASA PS (ASA I : II : III)	85 : 37 :14	
Injuries		
Mechanism of injury (blunt : penetrating)	118 : 18	
NISS	27 (1–75)	
ISS	19 (1–75)	
Serious injury (NISS ≥ 16; ISS ≥ 16)	92; 80	
Critical injury (NISS ≥ 25; ISS ≥ 25)	75; 57	
Major head injury^b^ (yes : no)	59 : 77	
Admission BE (mmol/L)	–3.1 (–26.0–3.4); *n* = 127	
Admission BE ≤ –6 mmol/L (*n*)	32	
TCC analyses		
Admission TCC (AU/mL)	0.87 (0.08–14.1); *n* = 136	0.42 (0.08–1.52)
TCC-AUC_3 − 6_ (AU/mL×h)	2.49 (0.25–52.6); *n* = 120	
Time from injury to first sample (hours : minutes)	1:15 (0:20–5:40)	
Samples analysed per patient	6 (1–35)	
Mean interpolated TCC (AU/mL)		
Day 0	0.88 (0.17–15.1); *n* = 136	
Day 4	1.91 (0.08–12.7); *n* = 39	
Day 7	2.24 (0.72–15.2); *n* = 28	
Day 9	2.39 (0.53–21.4); *n* = 23	
Hospital treatment		
Transfused before ICU (yes : no : unknown)	30 : 105 : 1	
Transfusions before ICU (PRBC units)	9 (1–61); *n* = 30	
Hospital length of stay (days)	6 (1–52)	
ICU length of stay	3 (1–52); *n* = 131	
SOFA score		
Day 0	6 (0–16); *n* = 133	
Day 4	9 (1–15); *n* = 39	
Day 7	9 (1–14); *n =* 26	
Day 9	9 (0–15); *n =* 20	
Ventilator treatment (yes : no)	67 : 69	
Survival		
Dead at 30 days (yes : no)	20 : 116	
Time to death (days)	1 (0–23); *n =* 20	
Predefined outcome variable		
Ventilator–free days	29 (0–30)	

Values are given as median and range unless otherwise stated

^a^ *n* is given where group size is < 136

^b^ Major head injury was defined as maximum Abbreviated Injury Scale (AIS) severity code ≥ 3 in ISS region Head or neck

*ASA PS* American Society of Anesthesiologists Physical Status Classification System, *NISS* New Injury Severity Score, *ISS* Injury Severity Score, *BE* Base Excess, *AU* Arbitrary units, *ICU* Intensive Care Unit, *PRBC* Packed Red Blood Cells, *SOFA* score Sequential Organ Failure Assessment score

### Complement concentration kinetics after trauma

**Fig. 1:**
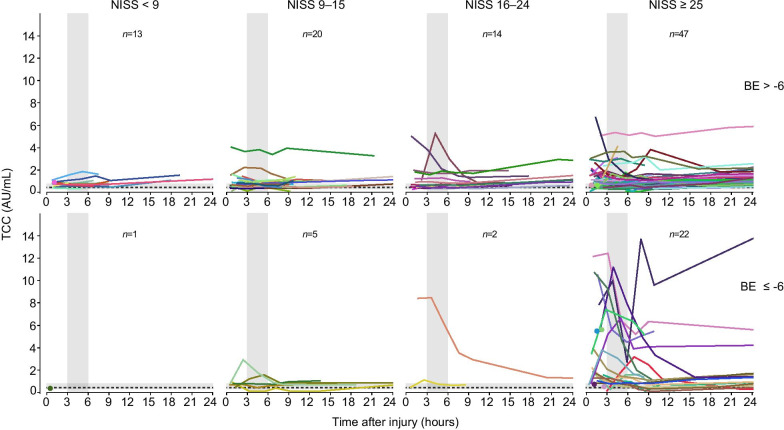
Complement activation during the first 24 hours after trauma. Individual Terminal Complement Complex (TCC) concentration curves for the 124 trauma patients with documented admission base excess (BE), grouped by severity of anatomical injury (New Injury Severity Score, NISS) and physiological derangement (BE). Black dashed lines represent median TCC concentration and grey corresponding areas interquartile ranges for the reference population of 20 healthy volunteers. The grey shaded columns show the time interval used for computation of area under the TCC concentration curve 3–6 hours after injury (TCC-AUC_3–6_)

**Fig. 2:**
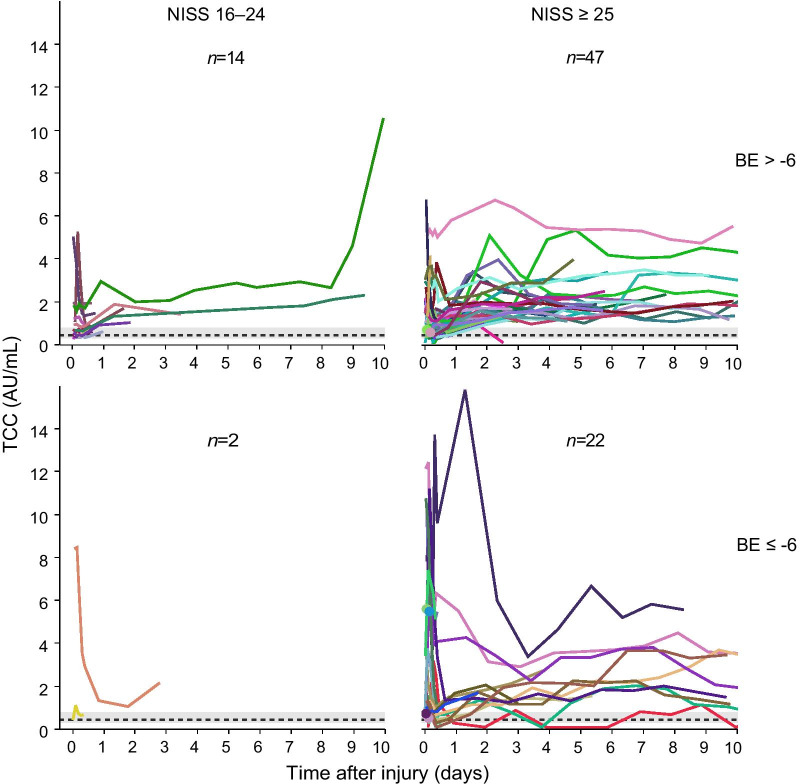
Complement activation during the first 10 days after trauma. Individual Terminal Complement Complex (TCC) concentration curves 0–10 days after injury for the 136 trauma patients constituting the study population. Black dashed lines represent median TCC concentration and grey corresponding areas interquartile range for the reference population of 20 healthy volunteers. Individual patients’ concentration curves are drawn in the same color as in Fig. 1

**Fig. 3:**
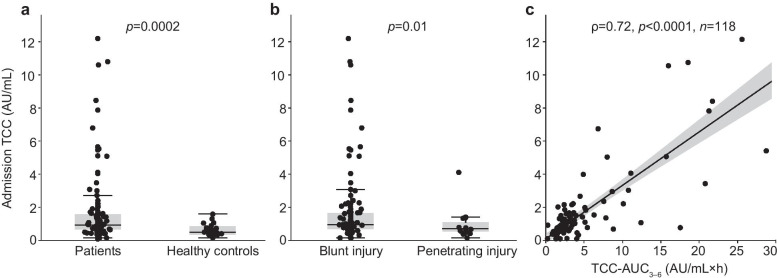
Admission TCC in patients and controls and relation to mechanism of injury. **a** Distribution of admission Terminal Complement Complex (TCC) in patients (*n=*133) and healthy controls (*n=*20). **b** Distribution of admission TCC values in patients with blunt (*n=*116) and penetrating (*n=*17) injury. **c** Admission TCC values plotted against TCC-AUC_3–6_ (*n*=118), with linear fit and 95% confidence interval for the fitted line (shaded). *ρ*, Spearman’s rank correlation coefficient

TCC concentration curves were heterogeneous among the individual trauma patients. Complement was already activated on admission and TCC concentrations were generally highest within the first 6 h after injury (Figs. 1 and 2). Admission TCC in the study population (*n* = 136) ranged from 0.08 to 14.1 AU/mL with median (quartiles) concentration 0.87 AU/mL (0.60–1.57). Admission TCC was significantly higher among patients (median [quartiles; range] 0.85 AU/mL [0.59–1.50; 0.08–12.14], *n =* 133, the three extreme outliers removed) than among the healthy volunteers (0.42 AU/mL [0.27–0.79; 0.08–1.52], *n =* 20) (*p* = 0.0002) (Fig. 3a). Admission TCC was higher in blunt (0.89 AU/mL [0.62–1.59; 0.08–12.14], *n* = 116) than penetrating (0.63 AU/mL [0.44–0.99; 0.08–4.01], *n* = 17) injury (*p* = 0.01, Fig. 3b), increased with both increasing NISS (*ρ* = 0.31, *p* = 0.0003) and decreasing BE (*ρ* = -0.21, *p* = 0.02) (Table S2 in Additional file 5), and was not statistically different between males and females (median 0.76 AU/mL vs. 0.93 AU/mL, respectively, *p* = 0.52).

Individual TCC concentration curves varied substantially from admission through day 10 after trauma (Fig. 2). We first investigated individual responses during the initial 24 h, for which a pattern emerged when TCC concentration curves were stratified by NISS and admission BE (Fig. 1). The impact of complement activation on the immune system was presumed to be a function not only of admission concentration but also concentration over time. In order to study this, we calculated area under the individual TCC concentration curve during the time period from 3 to 6 h after injury (TCC-AUC_3–6_) (Methods in Additional file 1 and illustrated as grey columns in Fig. 1). TCC-AUC_3–6_ was median (quartiles; range) 2.49 AU/mL×h (1.84–4.23; 0.25–52.6) (Table 1). TCC-AUC_3–6_ increased with increasing admission TCC (*ρ* = 0.72, *p* < 0.0001) (Fig. 3c) and NISS (*ρ* = 0.21, *p* = 0.02), and with decreasing BE (*ρ* = − 0.30, *p* = 0.001) (Table S2 in Additional file 5), but was not affected by injury mechanism (blunt median 2.47 AU/mL vs. penetrating 2.78 AU/mL, *p* = 0.87) or sex (males median 2.41 AU/mL vs. females 3.20 AU/mL, *p* = 0.35).

### Complement activation and outcome

**Fig. 4:**
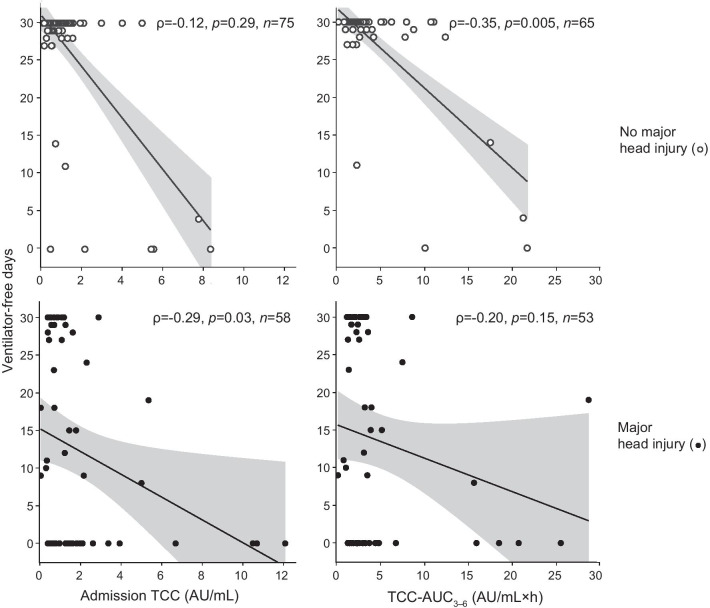
Ventilator-free days. Terminal Complement Complex (TCC) vs. ventilator-free days (VFD) for patients without major head injury (open symbols) and with major head trauma (filled symbols). Admission TCC concentrations are shown in the two left panels and TCC-AUC_3–6_ in the two right panels, with linear fit and 95% confidence interval for the fitted line (shaded). *ρ*, Spearman's rank correlation coefficient

**Fig. 5:**
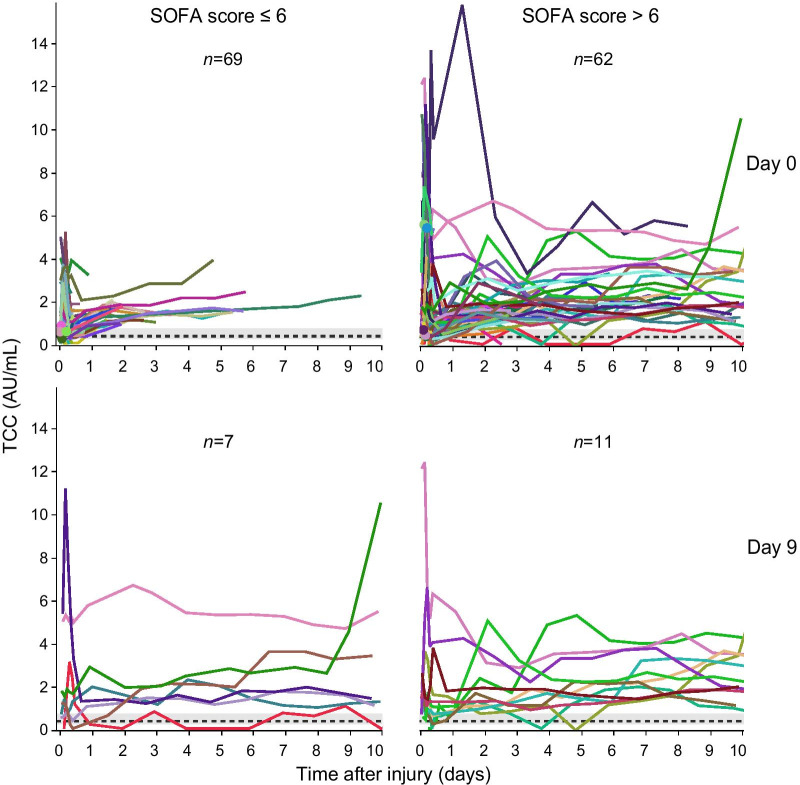
SOFA score. Total complement complex (TCC) concentration curves for 10 days for patients with low (≤ 6) and high (> 6) Sequential Organ Failure Assessment (SOFA) score at day 0 and 9 after trauma. Black dashed lines represent median TCC concentration and grey corresponding areas interquartile ranges for the reference population of 20 healthy volunteers

**Fig. 6:**
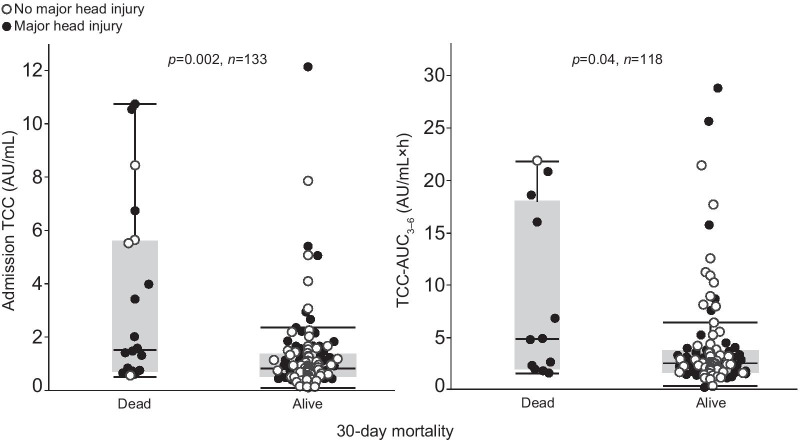
Mortality. Distribution of admission TCC and TCC-AUC_3–6_ for patients who died within 30 days and patients who survived. A total of 20 patients died, 16 of them due to major head injury. All patients who died had admission TCC documented, but TCC-AUC_3–6_ could be calculated in only 12 of them due to early deaths (see Figure S2 in Additional file 4). Open symbols represent patients without major head trauma; filled symbols represent patients with major head trauma. Values in the panels are numbers (*n*) and *p*-values for the total population

For our primary outcome measure VFD, observations clustered at the extremes of the scale (0 and 30 days, Fig. 4). In non-parametric bivariable analyses for the total population (Table S2 in Additional file 5) both admission TCC and TCC-AUC_3−6_ were correlated with VFD. Patients with low SOFA score (≤ 6) at the day of admission appeared to have lower admission TCC concentrations than patients with high SOFA score (> 6), Fig. 5. At day 9 however, there was apparently no difference between TCC concentration curves in patients with low and high SOFA score. In regression analyses, this apparent trend was confirmed; admission TCC, TCC-AUC_3−6_, and same-day TCC measurements were correlated with the secondary outcome SOFA score on day 0 and 4 after injury, but not on day 7 and 9 (Fig. S3 in Additional file 6). Both admission TCC and TCC-AUC_3−6_ were higher in patients who died (median 1.52 AU/mL vs. 0.81 AU/mL, *p* = 0.002 and 4.86 AU/mL vs. 2.43 AU/mL, *p =* 0.04 respectively) (Fig. 6).

### Multivariable analyses

Simultaneous relationships between patient and injury characteristics, complement activation, and outcome were explored in multivariable linear regression analyses (Tables S3 and S4 in Additional files 7 and 8). In the optimal model, admission TCC was associated with blunt mechanism of injury and decreasing admission BE, and to a lesser extent by increasing NISS (R^2^ = 0.37). TCC-AUC_3–6_ was associated with admission TCC alone (R^2^ = 0.64). VFD was associated with NISS and admission TCC (R^2^ = 0.64), without any contribution from sex, age, injury mechanism and admission BE. For daily SOFA scores (Table S4 in Additional file 8), NISS and TCC-AUC_3–6_ were the only associated variables, and only on day 0 and 4 (R^2^ = 0.60 and 0.58 respectively). All models were highly significant (*p* < 0.0001).

### Effects of major head injury

Major head injury is a main determinant for outcome after trauma. In this study, patients with and without major head injury had 30-day mortality of 37 % and 5 % (*p* = 0.0005) respectively, and median (quartiles; range) VFD of 9 (0–28; 0–30) and 30 (29–30; 0–30) (*p* < 0.0001), respectively (Table S1 in Additional file 3). Variables associated with outcome for the two subgroups were therefore also analysed separately (Fig. 4 and Fig. S3 in Additional file 6, and Tables S2, S3, S4 in Additional files 5, 7 and 8).

In multivariable models, VFD was associated with NISS alone in patients with major head injury (R^2^ = 0.52) and by TCC-AUC_3–6_, admission TCC, and NISS in those without (R^2^ = 0.59). NISS contributed to SOFA score on Day 0 for both subgroups, together with same-day TCC in those with major head injury (R^2^ = 0.42) and TCC-AUC_3–6_ in those without (R^2^ = 0.41). On Day 4, admission TCC was the sole variable associated with SOFA score in those with major head injury (R^2^ = 0.33), while NISS and TCC-AUC_3–6_ were associated in those without (R^2^ = 0.90). There was insufficient data to establish covariates associated with SOFA score on Day 7 and 9 in either subgroup. All other models were highly significant (*p* ≤ 0.002).

## Discussion

This study shows that plasma concentrations of TCC are elevated following trauma, both in the initial hours and in the succeeding days. Moreover, patients with more severe injury characteristics, i.e. higher NISS and lower admission BE, have higher levels of TCC both at admission and accumulated 3–6 h after injury. Here, we explore individual TCC concentration curves and show that although TCC curves exhibit great variability in temporal shapes between individuals, there is a pattern; the highest values were generally seen within the first 6 h after trauma, before TCC levels subside to a slightly lower level but remain elevated throughout the ICU stay. We found that complement activation 3 to 6 h after admission is a better predictor of multiple organ dysfunction syndrome than admission TCC. Thus, this time period should be in focus in the design of future experimental studies and clinical trials using complement inhibitors.

The heterogeneity observed may be caused by diversity in patient background, trauma characteristics and treatment, and moreover by the complement system itself. It is dynamic and volatile after an enormous impact such as major trauma, and it is only to be expected that the end product of the complement cascade, TCC, varies in both concentrations and timing among individuals. Thus, although complement activation products have short half-lives in plasma, the downstream tissue consequences of complement activation may go on in the organs for days and weeks (Huber-Lang et al. 2018).

Multivariable analyses are useful to explore the relative importance of explanatory variables. We found blunt injury and deranged physiology to be the most important explanatory variables for admission TCC, with a smaller contribution from anatomical injury. Admission TCC in turn predicted TCC-AUC_3–6_. We regard these data to fit well into our main hypothesis of the role of complement in the pathophysiology of remote organ dysfunction after severe trauma.

Complement activation was consistently associated with outcome. Both admission TCC and TCC-AUC_3–6_ were important explanatory variables for the primary outcome VFD in patients without major head trauma, together explaining more than 73 % of its variability. Moreover, TCC-AUC_3–6_ together with NISS were the only predictors for our secondary outcome SOFA score at day 0 and 4, in the same subgroup. No association was found between complement activation and SOFA score at day 7 and 9. This may be caused by our relatively small sample of patients with longer ICU stays, or possibly indicate that remote organ dysfunction later in the ICU stay is influenced by other or additional factors. Alternatively, it may indicate that complement activation as measured by TCC in plasma is less important for remote organ dysfunction than complement activation locally in the tissues (Huber-Lang et al. 2018).

We found an association between complement activation and mortality in this study, however most deaths occurred early from unsurvivable head injuries. Independent of the cause of death, complement activation was significantly higher among the deceased and one can speculate if it is a contributing trigger, or a marker of the following event.

A substantial number of papers exist on complement activation after trauma with two or more serially collected samples per patient (Risberg et al. 1986; Fosse et al. 1987, 1998; Zilow et al. 1992; Donnelly et al. 1994; Meade et al. 1994; Roumen et al. 1995; Hecke et al. 1997; Amara et al. 2009; Burk et al. 2012; Li et al. 2019), the highest time resolution studied previously being samples every 4 h (Amara et al. 2009; Burk et al. 2012). Our study contributes with an even higher time resolution with samples every 2 h for the first 5 samples. Further, despite many studies having a repeated-measures design, to our knowledge, ours is the first to utilise this design by presenting and analysing complement concentration kinetics in individual patients for up to 10 days after trauma. Studies utilising a repeated-measures design may have a better potential to answer clinically relevant questions, e.g. by generation of summary measures of time-varying data which can often be sufficiently analysed by simple statistical methods (Matthews et al. 1990). Our summary measure, TCC-AUC_3–6_, outperformed admission TCC as a predictor both for VFD and for SOFA scores on day 0 and 4 in patients without major head injury. This is hardly surprising, as it encompasses TCC concentration over time during 3 critical hours early after trauma (Karasu et al. 2019; Groeneveld et al. 2011).

Our study has several limitations. First, an even higher sampling time resolution during an extended time period and a higher number of patients would probably discriminate patterns of TCC concentration curves better. Second, to include mechanism of injury as an explanatory variable in our multivariable analyses can be debated, as there were only 13 % penetrating injuries in our population. Third, NISS as measure for injury severity scoring is not an accurate measure of total volume of tissue injury. Finally, the use of multivariable linear models when studying explanatory variables may be error prone, in particular when relationships between variables are suspected to be nonlinear, or when there are outliers. However, we did explore a non-linear approach with GAM, but this did only marginally influence the results.

## Conclusions

The present data demonstrates that complement was activated early after trauma, generally reached its highest concentration within 6 h of injury, and remained elevated in ICU patients for several days after injury. The degree of complement activation in the first critical hours was associated with blunt injury, deranged physiology and high NISS. In patients without major head injury, TCC-AUC_3–6_ outperformed admission TCC as a predictor for both VFD and SOFA score at day 0 and 4. Thus, the greatest surge of complement activation is found within the first 6 h after injury. This time period should be in focus when designing future experimental and clinical studies using complement inhibitors in the treatment of trauma.

## Supplementary Information

**Additional file 1.** Methods.

**Additional file 2: Figure S1.** STROBE flow diagram.

**Additional file 3: Table S1.** Characteristics of trauma patients with and without major head injury. 

**Additional file 4. Figure S2. **Time to death.

**Additional file 5. Table S2.** Correlation analyses.

**Additional file 6. Figure S3.** SOFA score versus TCC in patients with and without major head injury.

**Additional file 7. Table S3.** Regression analyses for ventilator-free days.

**Additional file 8. Table S4. **Regression analyses for SOFA scores.

**Additional file 9. Figure S4.** Assessment of SOFA scores. 

## Data Availability

The data that support the findings of this study are available from Oslo University Hospital, but restrictions to their availability apply. The data belong to OUH and are subject to Norwegian legislation regarding hospital owned data.

## Abbreviations

AIS: Abbreviated Injury Scale
AU: Arbitrary units
BE: Base excess
BIC: Bayesian information criterion
C3: Complement component 3
C3a: Complement component 3a
C5: Complement component 5
C5a: Complement component 5a
C5b-9: Complement component complex 5b-9
ELISA: Enzyme-linked immunosorbent assay
ICU: Intensive care unit
ISS: Injury Severity Score
K_2_EDTA: Dipotassium ethylenediaminetetraacetic acid
GAM: Generalised additive models
MAC: Membrane attack complex
MODS: Multiple organ dysfunction syndrome
NISS: New Injury Severity Score
OUH: Oslo University Hospital
SOFA score: Sequential Organ Failure Assessment score
STROBE: Strengthening the Reporting of Observational Studies in Epidemiology
TCC: Terminal complement complex
TCC-AUC_3–6_: Area under the individual TCC curve 3–6 hours after injury
VFD: Ventilator-free days
