# Supplementary material for: Increased complement activation 3 to 6 h after trauma is a predictor of prolonged mechanical ventilation and multiple organ dysfunction syndrome: a prospective observational study
Source: Mol Med. 2021 Apr 8;27:35. doi: 10.1186/s10020-021-00286-3 (PMC8028580; doi:10.1186/s10020-021-00286-3)
Supplement: Supplementary file 1 — Additional file 1. Methods. [file 10020_2021_286_MOEM1_ESM.pdf]

SUPPLEMENTAL METHODS

Sequential Organ Failure Assessment (SOFA) score

Data for SOFA scoring [1] was obtained from intensive care unit (ICU) patient records in paper and electronic form. The time interval for SOFA scoring at day 0 was defined as the 24-hour period starting at hospital arrival. Day 0 was defined as the calendar day of admission, unless the patient arrived ≤ 3 hours before midnight. If so, day 0 was set to the following calendar day. The definition was pragmatic to be able to define consecutive days. SOFA scores from day 4, 7, and 9 were obtained from the 24-hour periods starting 6 am at corresponding dates. **Supplemental Figure 4 in Additional file 9** gives information on how SOFA scores for the individual organs were defined, and further details on how our measure for respiratory function was obtained. Glasgow Coma Scale (GCS) score at day 0 was documented as the worst value without sedation in prehospital or inhospital records. If the patient was sedated, the most recent unsedated GCS value was used for SOFA scoring until unsedated GCS was assessed again. Where GCS was missing as a value in patient records, GCS was inferred from all information available, and the best possible value was recorded.

Calculation of area under individual TCC concentration curves (TCC $AUC_{3–6}$) and mean interpolated TCC (Daily TCC)

Concentrations of TCC were linearly interpolated with 7.5 minutes time resolution from the time of injury. This enabled analysis mandating comparison of data at specific times after injury across our asynchronously sampled data. The linearly interpolated data set was also used to calculate area under the individual TCC curves during the 3-hour interval starting 3 hours after trauma (TCC $AUC_{3–6}$) using the trapezoidal rule, and to assess mean interpolated TCC (Daily TCC) at day 0, 4, 7 and 9. The application for linear interpolation followed by integration was

custom developed in LabVIEW 2013 (National Instruments, Austin, TX). The time interval 3–6 hours after injury was utilised since it covers a major part of the large peaks in the TCC curves, and moreover, because at 3 hours 127 of the 136 patients (93%) were already admitted and at 6 hours 16 of the 20 patients who died were still alive and only four patients already discharged, leaving a total of 119 patients (88%) observable. Furthermore, this time interval seems prognostically relevant in analyses of the alarmin HMGB1 after trauma [2]. Mean interpolated TCC (Daily TCC) was chosen to represent average concentration of TCC on a given day. Days 0, 4, 7 and 9 were chosen because SOFA scores were available for those days.

REFERENCES

1. Vincent JL, Moreno R, Takala J, Willatts S, De Mendonca A, Bruining H, Reinhart CK, Suter PM, Thijs LG: **The SOFA (Sepsis-related Organ Failure Assessment) score to describe organ dysfunction/failure. On behalf of the Working Group on Sepsis-Related Problems of the European Society of Intensive Care Medicine**. *Intensive Care Med* 1996, **22**(7):707-710.
2. Ottestad W, Rognes IN, Pischke SE, Mollnes TE, Andersson U, Eken T: **Biphasic Release of the Alarmin High Mobility Group Box 1 Protein Early After Trauma Predicts Poor Clinical Outcome**. *Crit Care Med* 2019, **47**(8):e614-e622.
