# Supplementary material for: Increased complement activation 3 to 6 h after trauma is a predictor of prolonged mechanical ventilation and multiple organ dysfunction syndrome: a prospective observational study
Source: Mol Med. 2021 Apr 8;27:35. doi: 10.1186/s10020-021-00286-3 (PMC8028580; doi:10.1186/s10020-021-00286-3)
Supplement: Supplementary file 2 — Additional file 2: Figure S1. STROBE flow diagram. [file 10020_2021_286_MOEM2_ESM.pdf]

STROBE flow diagram for patient inclusion, blood samples analysed for TCC and statistical analyses

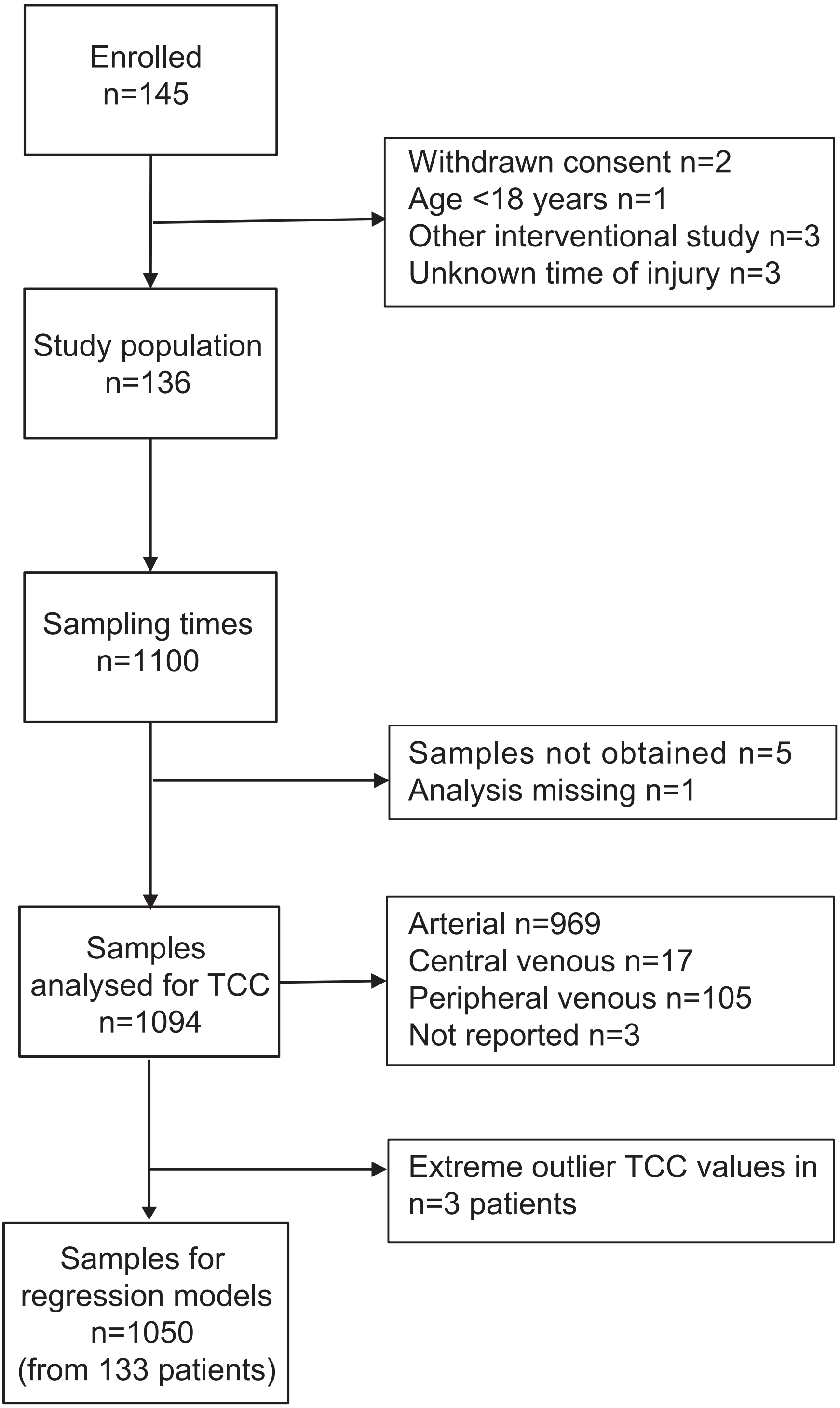

Supplemental Figure 1. STROBE flow diagram
Flow diagram for the study participants and TCC analyses, according to von Elm E, Altman DG, Egger M et al.: The Strengthening for Reporting of Observational Studies in Epidemiology (STROBE) statement: Guidelines for reporting observational studies. Lancet 2007;370:1453–1457.
