# Supplementary material for: Increased complement activation 3 to 6 h after trauma is a predictor of prolonged mechanical ventilation and multiple organ dysfunction syndrome: a prospective observational study
Source: Mol Med. 2021 Apr 8;27:35. doi: 10.1186/s10020-021-00286-3 (PMC8028580; doi:10.1186/s10020-021-00286-3)
Supplement: Supplementary file 3 — Additional file 3: Table S1. Characteristics of trauma patients with and without major head injury. [file 10020_2021_286_MOEM3_ESM.pdf]

Supplemental Table 1. Characteristics of trauma patients with and without major head injury

Characteristics	No Major Head Injury[a] (*n*=77)[b]	Major Head Injury[a] (*n*=59)[b]	*p*
**Demographics**			
Sex (male : female)	57 : 20	44 : 15	1.00
Age (years)	37 (18 – 75); *n*=76	42 (18 – 94); *n*=59	0.17
Preinjury ASA PS (ASA I : II : III)	54 : 18 : 5	31 : 19 : 9	0.08
**Injuries**			
Mechanism of injury (blunt : penetrating)	66 : 11	52 : 7	0.80
NISS	14 (1 – 59)	48 (10 – 75)	***<0.0001***
ISS	10 (1 – 43)	34 (9 – 75)	***<0.0001***
Admission BE (mmol/L)	-3.0 (-25.9 – 3.4); *n*=70	-3.9 (-26.0 – 2.1); *n*=57	0.25
**TCC analyses**			
Admission TCC (AU/mL)	0.81 (0.08 – 14.1)	1.11 (0.08 – 12.1)	0.08
TCC-AUC$_{3–6}$ (AU/mL×h)	2.38 (0.28 – 21.8); *n*=66	2.94 (0.25 – 52.6); *n*=54	0.29
Time from injury to first sample (hours: min)	1:07 (0:20 – 4:06)	1:23 (0:28 – 5:40)	***0.02***
Time from adm. to first sample (hours: min)	0:10 (0:00 – 0:53)	0:10 (0:00 – 1:27)	0.63
Samples analysed per patient	6 (1 – 35)	8 (1 – 29)	***<0.0001***
Mean interpolated TCC (AU/mL)			
Day 0	0.80 (0.17 – 15.1); *n*=77	1.04 (0.30 – 7.04); *n*=59	0.05
Day 4	1.68 (0.47 – 12.7); *n*=13	1.91 (0.08 – 5.39); *n*=26	0.91
Day 7	2.65 (1.54 – 15.2); *n*=8	2.24 (0.72 – 5.05); *n*=20	0.42
Day 9	2.68 (1.34 – 21.4); *n*=6	2.39 (0.53 – 7.64); *n*=17	0.62
**Hospital treatment**			
Primary : secondary admission	73 : 4	47 : 12	***0.01***
Time from injury to admission (hours)	0:52 (0:10 – 3:50)	1:05 (0:13 – 5:25)	0.06
Transfused before ICU (yes : no : unknown)	11 : 65 : 1	19 : 40 : 0	***0.02***
Transfusions before ICU (RBC units)	11 (2 – 24); *n*=11	4 (1 – 61); *n*=19	0.17
Hospital length of stay (days)	5 (1 – 50)	8 (1 – 52)	***0.02***
ICU length of stay (days)	2 (1 – 35); *n*=74	6 (1 – 52); *n*=57	***0.0008***
SOFA score			
Day 0	3 (0 – 13); *n*=74	9 (1 – 16); *n=59*	***<0.0001***
Day 4	3 (1 – 15); *n*=12	9 (4 – 15); *n*=27	0.08
Day 7	10 (1 – 14); *n*=7	8 (4 – 12); *n*=19	0.38
Day 9	10 (0 – 15); *n*=5	8 (1 – 10); *n*=15	0.19
Ventilator treatment (yes : no)	20 : 57	47 : 12	***<0.0001***
Time on ventilator (days)	2 (1 – 26); *n*=20	6 (1 – 35); *n*=47	0.07
Sent to other hospital still intubated (yes : no)	3 : 17	22 : 25	***0.02***
**Survival**			
Dead at 30 days (yes : no)	4 : 73	16 : 43	***0.0005***
Time to death (days)	0 (0 – 3); *n*=4	1 (0 – 23); *n*=16	0.31
**Predefined outcome variable**			
Ventilator-free days	30 (0 – 30)	9 (0 – 30)	***<0.0001***

Values are given as median and range unless otherwise stated.

[a] Major head injury was defined as maximum Abbreviated Injury Scale (AIS) severity code ≥3 in ISS region Head or neck.

[b] *n* is given only where group size is <59 or <77.

Abbreviations: ASA PS = American Society of Anesthesiologists Physical Status Classification System. NISS = New Injury Severity Score. ISS = Injury Severity Score. BE = Base Excess. AU = Arbitrary units. Adm. = Admission. ICU = Intensive Care Unit. (RBC = Packed Red Blood Cells. SOFA score = Sequential Organ Failure Assessment score.
