# Supplementary material for: Increased complement activation 3 to 6 h after trauma is a predictor of prolonged mechanical ventilation and multiple organ dysfunction syndrome: a prospective observational study
Source: Mol Med. 2021 Apr 8;27:35. doi: 10.1186/s10020-021-00286-3 (PMC8028580; doi:10.1186/s10020-021-00286-3)
Supplement: Supplementary file 4 — Additional file 4. Figure S2. Time to death. [file 10020_2021_286_MOEM4_ESM.pdf]

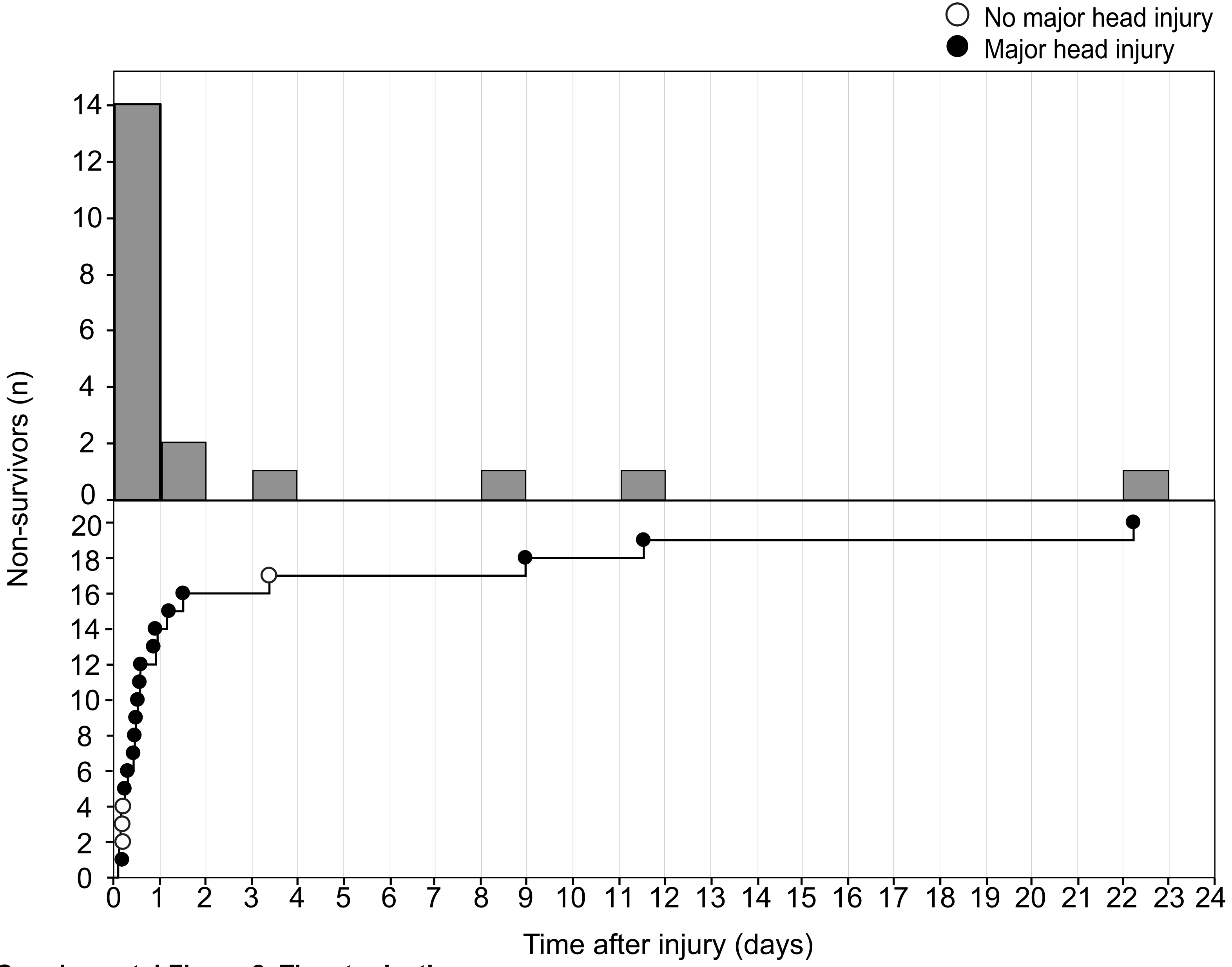

Supplemental Figure 2. Time to death

Time to death for all deceased patients (lower panel), with number of deaths per day (24-hour period) (upper panel). Only four of the patients who died did not have major head injury. 16 patients died during the first 48 hours after trauma, nine of them from major traumatic brain injury and five of them from massive haemorrhage.
