# Supplementary material for: Increased complement activation 3 to 6 h after trauma is a predictor of prolonged mechanical ventilation and multiple organ dysfunction syndrome: a prospective observational study
Source: Mol Med. 2021 Apr 8;27:35. doi: 10.1186/s10020-021-00286-3 (PMC8028580; doi:10.1186/s10020-021-00286-3)
Supplement: Supplementary file 5 — Additional file 5. Table S2. Correlation analyses. [file 10020_2021_286_MOEM5_ESM.pdf]

Supplemental Table 2. Bivariable analyses – Non-parametric Spearman correlation

Characteristics	All Trauma Patients		No Major Head Injury[a]		Major Head Injury[a]	
	ρ (*n*)[b]	*p*-value	***ρ*** (*n*)[b]	*p*-value	***ρ*** (*n*)[b]	*p*-value
**Admission TCC (AU/mL)**	*n*=133		*n*=75		*n*=58	
Age (years)	0.14 (*132*)	0.11	0.17 (*74*)	0.16	0.07	0.62
Admission BE (mmo/L)	-0.21 (*124*)	***0.02***	-0.13 (*68*)	0.31	-0.28 (*56*)	***0.04***
NISS	0.31	***0.0003***	0.18	0.12	0.31	0.02
**TCC-AUC$_{3–6}$ (AU/mL×h)**	*n*=118		*n*=65		*n*=53	
Age (years)	0.07 (*117*)	0.46	0.07 (*64*)	0.59	0.06	0.67
Admission BE (mmo/L)	-0.30 (*110*)	***0.001***	-0.17 (*59*)	0.21	-0.42 (*51*)	***0.002***
NISS	0.21	0.02	0.13	0.31	0.23	0.10
Admission TCC (AU/mL)	0.72	***<0.0001***	0.67	***<0.0001***	0.80	***<0.0001***
**Ventilator Free Days**	*n*=133		*n*=75		*n*=58	
Age (years)	-0.14 (*132*)	0.12	-0.05 (*74*)	0.65	-0.05	0.72
Admission BE (mmo/L)	0.44 (*124*)	***<0.0001***	0.39 (*68*)	***0.001***	0.46 (*56*)	***0.0003***
NISS	-0.75	***<0.0001***	-0.43	***0.0001***	-0.72	***<0.0001***
Admission TCC (AU/mL)	-0.28	***0.001***	-0.12	0.29	-0.29	***0.03***
TCC-AUC$_{3–6}$ (AU/mL×h)	-0.28 (*118*)	***0.002***	-0.35 (*65*)	***0.005***	-0.20 (*53*)	0.15
**SOFA score Day 0**	*n*=130		*n*=72		*n*=58	
Age (years)	0.13 (*129*)	0.13	0.19 (*71*)	0.11	-0.09	0.51
Admission BE (mmo/L)	-0.47 (*121*)	***<0.0001***	-0.40 (*65*)	***0.0009***	-0.56 (*56*)	**<0.0001**
NISS	0.70	***<0.0001***	0.49	***<0.0001***	0.59	***<0.0001***
Admission TCC (AU/mL)	0.27	***0.002***	0.15	0.21	0.33	***0.01***
TCC-AUC$_{3–6}$ (AU/mL×h)	0.34 (*118*)	***0.0002***	0.27 (*63*)	***0.03***	0.43 (*53*)	***0.001***
Daily TCC Day 0 (AU/mL)	0.35	***<0.0001***	0.32	***0.007***	0.34	0.008
**SOFA score Day 4**	*n*=37		*n*=11		*n*=26	
Age (years)	0.11	0.54	-0.24	0.48	0.04	0.86
Admission BE (mmo/L)	-0.30 (*35*)	***0.08***	-0.34 (*10*)	0.34	-0.17 (*25*)	0.43
NISS	0.64	***<0.0001***	0.63	***0.04***	0.51	***0.008***
Admission TCC (AU/mL)	0.44	***0.007***	0.63	***0.04***	0.37	0.06
TCC-AUC$_{3–6}$ (AU/mL×h)	0.44	***0.006***	0.69	***0.02***	0.23	0.26
Daily TCC Day 4 (AU/mL)	0.35 (*34*)	***0.04***	0.48	0.14	0.27 (*23*)	0.21
**SOFA score Day 7**	*n*=24		*n*=6		*n*=18	
Age (years)	-0.03	0.89	-0.09	0.87	-0.25	0.32
Admission BE (mmo/L)	-0.15 (*23*)	0.50	-0.56 (*5*)	0.32	0.005 (*18*)	0.98
NISS	0.30	0.16	0.75	0.08	0.20	0.43
Admission TCC (AU/mL)	0.16	0.47	0.55	0.26	-0.02	0.95
TCC-AUC$_{3–6}$ (AU/mL×h)	0.11	0.62	0.75	0.08	-0.31	0.21
Daily TCC Day 7 (AU/mL)	0.28	0.19	0.73	0.10	0.02	0.93
**SOFA score Day 9**	*n*=18		*n*=4		*n*=14	
Age (years)	-0.03	0.90	0.80	0.20	-0.31	0.28
Admission BE (mmo/L)	-0.09 (*17*)	0.73	0.50 (*3*)	0.67	-0.15 (*14*)	0.61
NISS	0.22	0.38	0.40	0.60	0.30	0.30
Admission TCC (AU/mL)	0.10	0.71	Too few data		-0.002	0.99
TCC-AUC$_{3–6}$ (AU/mL×h)	-0.08	0.75	0.40	0.60	-0.37	0.19
Daily TCC Day 9 (AU/mL)	0.17	0.50	Too few data		-0.02	0.93

[a] Major head injury was defined as maximum Abbreviated Injury Scale (AIS) severity code ≥3 in ISS region Head or neck.

[b] *n* is given where group size is less than given in each category heading.

ρ represent Spearman's rho and *p*-values represent two-tailed probability. Abbreviations: AU = Arbitrary units. SOFA score = Sequential Organ Failure Assessment score. BE = Base Excess. NISS = New Injury Severity Score.
