# Supplementary material for: Increased complement activation 3 to 6 h after trauma is a predictor of prolonged mechanical ventilation and multiple organ dysfunction syndrome: a prospective observational study
Source: Mol Med. 2021 Apr 8;27:35. doi: 10.1186/s10020-021-00286-3 (PMC8028580; doi:10.1186/s10020-021-00286-3)
Supplement: Supplementary file 6 — Additional file 6. Figure S3. SOFA score versus TCC in patients with and without major head injury. [file 10020_2021_286_MOEM6_ESM.pdf]

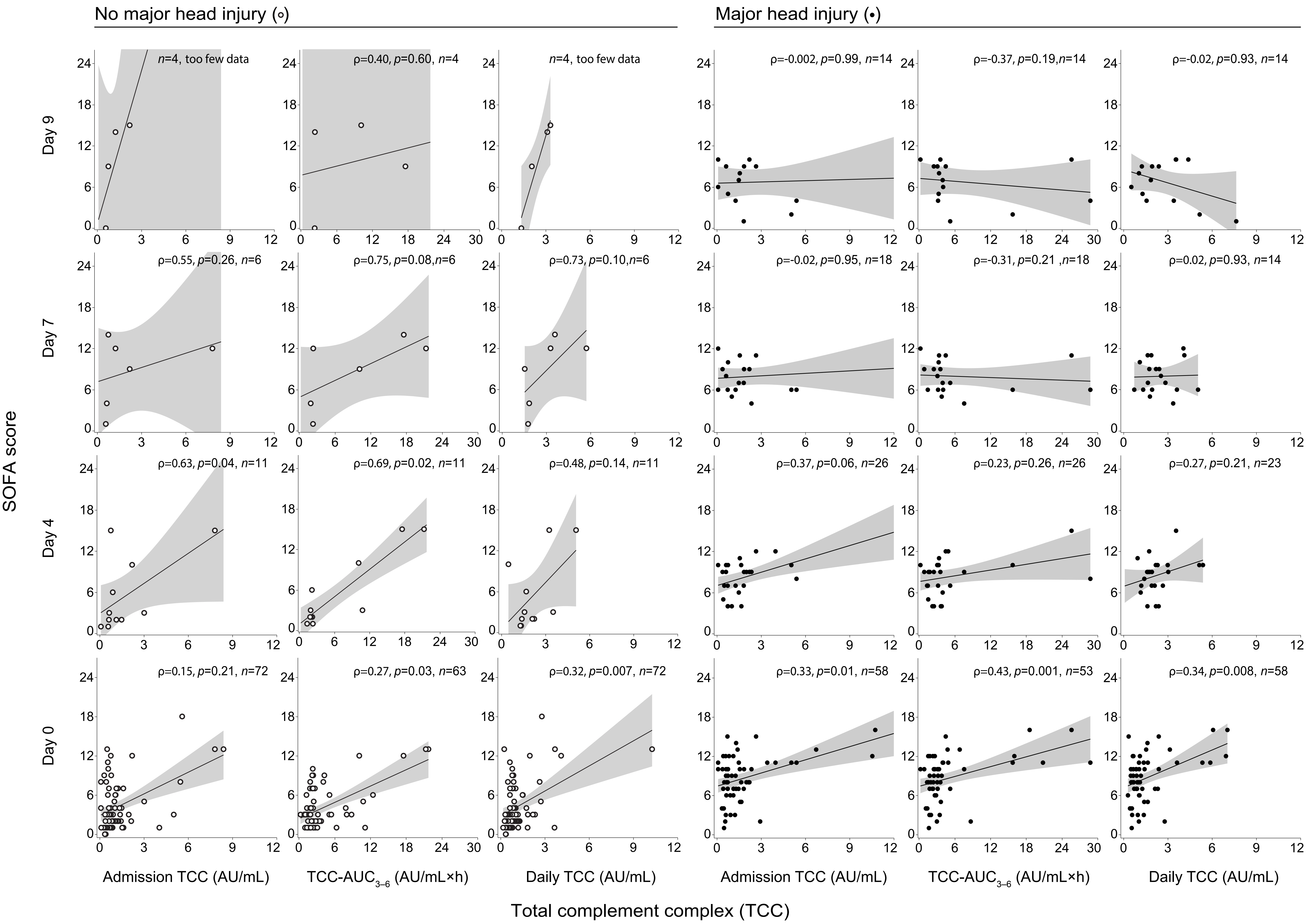

Supplemental Figure 3. SOFA score versus TCC.

Sequential Organ Failure Assessment (SOFA) score at day 0, 4, 7, and 9 after trauma, plotted against admission Terminal Complement Complex (TCC), TCC-AUC$_{3-6}$, and same-day TCC values. Open symbols represent patients without major head trauma; filled symbols represent patients with major head trauma. Data presented are numbers (*n*), Spearman's correlation coefficients (ρ) and p-values.
