# Supplementary material for: Increased complement activation 3 to 6 h after trauma is a predictor of prolonged mechanical ventilation and multiple organ dysfunction syndrome: a prospective observational study
Source: Mol Med. 2021 Apr 8;27:35. doi: 10.1186/s10020-021-00286-3 (PMC8028580; doi:10.1186/s10020-021-00286-3)
Supplement: Supplementary file 7 — Additional file 7. Table S3. Regression analyses for ventilator-free days. [file 10020_2021_286_MOEM7_ESM.pdf]

Supplemental Table 3. Univariate regressions and multivariable linear regression models for admission TCC, TCC-$AUC_{3\text{-}6}$ and ventilator-free days

Outcome	All Trauma Patients						No Major Head Injury[a]						Major Head Injury[a]					
Explanatory variable	Univariate regressions		Multivariable linear regression model				Univariate regressions		Multivariable linear regression model				Univariate regressions		Multivariable linear regression model			
	Est (95% CI)	p (R^2)	Est (95% CI)	V.I.	*n*[b]	P (R^2)	Est (95% CI)	p (R^2)	Est (95% CI)	V.I.	*n*[b]	p (R^2)	Est (95% CI)	p (R^2)	Est (95% CI)	V.I.	*n*[b]	p (R^2)
**Admission TCC**			***n*=133**						***n*=75**						***n*=58**			
Sex (M : F)	-0.30 (-0.70 – 0.10)	.15 (.02)					-0.39 (-0.79 – 0.01)	.06 (.05)					-0.18 (-0.95 – 0.58)	.64 (.004)				
Age (years)	0.01 (-0.01 – 0.03)	.16 (.02)			*132*		0.03 (0.004 – 0.05)	.02 (.07)			*74*		0.0008 (-0.03 – 0.03)	.96 ($5×10^{-5}$)				
MOI (B : P)	0.40 (-0.12 – 0.93)	.13 (.02)	0.72 (0.26 – 1.19)	.465		.003	0.20 (-0.32 – 0.71)	.45 (.008)	0.53 (0.03 – 1.04)	.548		.04	0.69 (-0.40 – 1.77)	.21 (.03)	0.99 (0.16 – 1.82)	.528		.02
NISS	0.04 (0.02 – 0.05)	<.0001 (.12)	0.02 (0.01 – 0.04)	.179		.01	0.04 (0.02 – 0.07)	.0008 (.14)					0.05 (0.009 – 0.09)	.02 (.10)				
Adm. BE	-0.21 (-0.28 – -0.15)	<.0001 (.28)	-0.21 (-0.27 – -0.15)	.356	*124*	<.0001	-0.13 (-0.20 – -0.07)	.0001 (.20)	-0.15 (-0.21 – -0.08)	.452	*68*	<.0001	-0.34 (-0.45 – -0.23)	<.0001 (.41)	-0.35 (-0.46 – -0.24)	.472	*56*	<.0001
Total model					*124*	<.0001 (.37)					*68*	<.0001 (.25)					*56*	<.0001 (.47)
**TCC-$AUC_{3–6}$**			***n*=118**						***n*=65**						***n*=53**			
Sex (M : F)	-0.82 (-1.92 – 0.29)	.14 (.02)					-0.69 (-1.98 – 0.59)	.28 (.02)					-0.94 (-2.88 – 0.99)	.33 (.02)				
Age (years)	0.02 (-0.03 – 0.08)	.41 (.006)			*117*		0.02 (-0.06 – 0.10)	.67 (.003)			*64*		0.02 (-0.07 – 0.11)	.63 (.005)				
MOI (B : P)	0.28 (-1.22 – 1.78)	.72 (.001)					-0.42 (-2.03 – 1.19)	.61 (.004)					1.34 (-1.59 – 4.26)	.36 (.02)				
NISS	0.07 (0.02 – 0.12)	.004 (.07)					0.15 (0.07 – 0.22)	.0004 (.18)	0.08 (0.03 – 0.13)	.212		.004	0.07 (-0.04 – 0.17)	.20 (.03)				
Adm. BE	-0.62 (-0.82 – -0.43)	<.0001 (.28)			*110*		-0.40 (-0.65 – -0.15)	.002 (.15)			*59*		-0.85 (-1.14 – -0.55)	<.0001 (.40)			*51*	
Adm. TCC	2.01 (1.74 – 2.29)	<.0001 (.64)	2.01 (1.74 – 2.29)			<.0001	2.36 (1.89 – 2.83)	<.0001 (.62)	2.17 (1.71 – 2.63)	.788		<.0001	1.92 (1.54 – 2.30)	<.0001 (.67)	1.92 (1.54 – 2.30)			<.0001
Total model					*118*	<.0001 (.64)					*65*	<.0001 (.66)					*53*	<.0001 (.67)
**Ventilator-free days**			***n*=133**						***n*=75**						***n*=58**			
Sex (M : F)	0.74 (-1.76 – 3.24)	.56 (.003)					1.17 (-0.99 – 3.33)	.28 (.02)					0.35 (-3.61 – 4.30)	.86 (.0005)				
Age (years)	-0.18 (-0.29 – -0.06)	.003 (.06)			*132*		-0.18 (-0.31 – -0.05)	.006 (.10)			*74*		-0.06 (-0.22 – 0.10)	.45 (.01)				
MOI (B : P)	-1.07 (-4.37 – 2.23)	.52 (.003)					0.08 (-2.64 – 2.80)	.95 (.00005)					-1.18 (-6.86 – 4.50)	.68 (.003)				
NISS	-0.50 (-0.57 – -0.43)	<.0001 (.61)	-0.46 (-0.53 – -0.39)	.761		<.0001	-0.35 (-0.47 – -0.24)	<.0001 (.35)	-0.15 (-0.25 – -0.06)	.266		.002	-0.57 (-0.72 – -0.43)	<.0001 (.52)	-0.57 (-0.72 – -0.43)			<.0001
Adm. BE	1.05 (0.66 – 1.45)	<.0001 (.19)			*124*		0.98 (0.72 – 1.24)	<.0001 (.46)			*68*		1.05 (0.38 – 1.72)	.003 (.15)			*56*	
Adm. TCC	-2.64 (-3.6 – -1.67)	<.0001 (.18)	-1.07 (-1.76 – -0.37)	.239		.003	-3.41 (-4.36 – -2.47)	<.0001 (.42)	-1.00 (-2.19 – 0.20)	.271		.10	-1.51 (-2.83 – -0.19)	.03 (.09)				
TCC-$AUC_{3–6}$	-0.80 (-1.20 – -0.40)	.0001 (.12)			*118*		-1.06 (-1.32 – -0.80)	<.0001 (.51)	-0.61 (-1.03 – -0.19)	.463	*65*	.005	-0.44 (-1.02 – 0.14)	.13 (.04)			*53*	
Total model					*133*	<.0001 (.64)					*65*	<.0001 (.59)					*58*	<.0001 (.52)

[a] Major head injury was defined as maximum Abbreviated Injury Scale (AIS) severity code ≥3 in Injury Severity Score region Head or neck.

[b] *n* is given where group size is less than *n* given in heading.

Abbreviations: V.I. = Variable Importance. M : F = Male : Female. MOI = Mechanism of injury. B : P = Blunt : Penetrating. NISS = New Injury Severity Score. Adm. = Admission. BE = Base Excess (mmol/L). *p* values represent two-tailed probability.
