# Supplementary material for: Increased complement activation 3 to 6 h after trauma is a predictor of prolonged mechanical ventilation and multiple organ dysfunction syndrome: a prospective observational study
Source: Mol Med. 2021 Apr 8;27:35. doi: 10.1186/s10020-021-00286-3 (PMC8028580; doi:10.1186/s10020-021-00286-3)
Supplement: Supplementary file 8 — Additional file 8. Table S4. Regression analyses for SOFA scores. [file 10020_2021_286_MOEM8_ESM.pdf]

Supplemental Table 4. Univariate regressions and multivariable linear regression models for SOFA scores

SOFA Score	All Trauma Patients					No Major Head Injury[a]					Major Head Injury[a]				
Explanatory variable	Univariate regressions		Multivariable linear regression model			Univariate regressions		Multivariable linear regression model			Univariate regressions		Multivariable linear regression model		
	Est (95% CI)	*p* / R^2	Est (95% CI)	V.I. (*n*)[b]	*p* / R^2	Est (95% CI)	*p* / R^2	Est (95% CI)	V.I. (*n*)[b]	*p* / R^2	Est (95% CI)	*p* / R^2	Est (95% CI)	V.I. (*n*)[b]	*p* / R^2
**Day 0**			***n*=130**					***n*=72**					***n*=58**		
Sex (M : F)	-0.38 (-1.22 – 0.45)	.36/.006				-0.62 (-1.55 – 0.31)	.19/.02				-0.12 (-1.21 – 0.97)	.83/.001			
Age	0.04 (-0.002 – 0.08)	.06/.03		*(129)*		0.06 (0.01 – 0.12)	.03/.07		*(71)*		-0.01 (-0.06 – 0.03)	.57/.01			
MOI (B : P)	0.22 (-0.87 – 1.30)	.70/.001				-0.06 (-1.21 – 1.10)	.92/.0001				0.01 (-1.55 – 1.58)	.99/.5×10^{-6}			
NISS	0.15 (0.12 – 1.18)	<.0001/.50	0.14 (0.11–0.16)	.730	<.0001	0.13 (0.08 – 0.18)	<.0001/.26	0.08 (0.03– 0.14)	.424	.004	0.13 (0.08 – 0.18)	.0001/.34	0.11 (0.06 –0.16)	.672	<.0001
Adm. BE	-0.41 (-0.54 – -0.27)	<.0001/.23		*(110)*		-0.33 (-0.48– -0.18)	<.0001/.23		*(65)*		-0.43 (-0.60 – -0.27)	.0001/.34		*(56)*	
Adm. TCC	0.90 (0.58 – 1.22)	<.0001/.19				0.86 (0.35 – 1.38)	.001/.14				0.67 (0.34 – 1.01)	.0002/.23			
TCC-AUC$_{3-6}$	0.35 (0.22 – 0.47)	<.0001/.21	0.22 (0.13–0.31)	.270	<.0001	0.42 (0.26 – 0.57)	<.0001/.33	0.32 (0.16– 0.48)	.576	.0002	0.25 (0.11 – 0.39)	.0009/.20			
Daily TCC (0)	1.24 (0.79 – 1.69)	<.0001/.19				1.13 (0.57 – 1.69)	.0001/.19				0.96 (0.42 – 1.50)	.0007/.19	0.67 (0.20 –1.15)	.328 *(53)*	.006
Total model				*(116)*	<.0001 /.60				*(63)*	<.0001 /.41				*(58)*	<.0001 /.42
**Day 4**			***n*=37**					***n*=11**					***n*=26**		
Sex (M : F)	-1.21 (-2.59 – 0.18)	.09/.08				-1.86 (-6.69 – 2.96)	.41/.08				-0.75 (-1.92 – 0.41)	.19./.07			
Age	0.03 (-0.04 – 0.10)	.38/.02		*(35)*		0.01 (-0.34 – 0.36)	.93/.0009		*(10)*		0.01 (-0.04 – 0.07)	.61./.01		*(25)*	
MOI (B : P)	0.26 (-2.66 – 3.18)	.86/.0009				–					0.73 (-1.41 – 2.87)	.49/.02			
NISS	0.17 (0.10 – 0.24)	<.0001/.41	0.15 (0.09–0.21)	.740	<.0001	0.37 (0.20 – 0.55)	.001/.72	0.18 (0.02 – 0.35)	0.526	.03	0.10 (0.03 – 0.16)	.005/.29			
Adm. BE	-0.38 (-0.67 – -0.10)	.009/.19				-0.96 (-1.88– -0.34)	.04/.42				-0.25 (-0.46 – -0.03)	.03/.19			
Adm. TCC	0.87 (0.39 – 1.36)	.0008/.28				1.46 (-0.05 – 2.97)	.06/.35				0.65 (0.26 – 1.04)	.002/.33	0.65 (0.26 – 1.04)		.002
TCC-AUC$_{3-6}$	0.28 (0.11 – 0.44)	.002/.25	0.23 (0.10–0.36)	.260	.0008	0.68 (0.43 – 0.92)	.0001/.81	0.46 (0.18– 0.74)	0.474	.005	0.14 (-0.02 – 0.29)	.08/.13			
Daily TCC (4)	1.35 (0.24 – 2.46)	.02/.16		*(34)*		2.27 (-0.33 – 4.87)	.08/.30				0.71 (-0.29 – 1.70)	.15/.09		*(23)*	
Total model				*(37)*	<.0001 /.58				*(11)*	.0001 / 0.90				*(26)*	.002 /.33
**Day 7**			***n*=24**					***n*=6**					***n*=18**		
Sex (M : F)	-0.7 (-2.08 – 0.68)	.31/.05				-2 (-10.26 – 6.26)	.54/.10				-0.61 (-1.81 – 0.58)	.29/.07			
Age	-0.01 (-0.08 – 0.06)	.77/.004				0.09 (-0.43 – 0.60)	.66/.05				-0.02 (-0.08 – 0.04)	.48/.03			
MOI (B : P)	1.16 (-1.27 – 3.59)	.33/.04				–					1.09 (-0.78 – 2.96)	.23/.09			
NISS	0.08 (-0.01 – 0.18)	.08/.13				0.28 (-0.03 – 0.59)	.06/.62				0.03 (-0.06 – 0.13)	.45/.04			
Adm. BE	-0.18 (-0.47 – 0.11)	.21/.08		*(23)*		-0.79 (-2.67 – 1.09)	.28/.37		*(5)*		-0.08 (-0.32 – 0.16)	.50/.03		*(18)*	
Adm. TCC	0.25 (-0.24 – 0.74)	.31/.05				0.69 (-1.64 – 3.03)	.46/.15				0.12 (-0.32 – 0.56)	.57/.02			
TCC-AUC$_{3-6}$	0.08 (-0.09 – 0.25)	.34/0.4				0.41 (-0.20 – 1.02)	.14/.46				-0.03 (-0.19 – 0.12)	.67/.01			
Daily TCC (7)	0.82 (-0.21 – 1.86)	.11/.11				2.15 (-1.15 – 5.44)	.15/.45				0.07 (-1.01 – 1.14)	.90/.001			
Total model			NF					NF					NF		
**Day 9**			***n*=18**					***n*=4**					***n*=14**		
Sex (M : F)	-0.20 (-2.37 – 1.98)	.85/.002				–					-0.86 (-2.64 – 0.93)	.32/.08			
Age[2]	-0.03 (-0.14 – 0.08)	.59/.02				0.40 (-1.24 – 2.03)	.40/.35				-0.06 (-0.14 – 0.02)	.14/.17			
MOI (B : P)	1.31 (-1.99 – 4.61)	.41/.04				–					1 (-1.59 – 3.59)	.42/.06			
NISS	0.10 (-0.04 – 0.25)	.16/.12				0.41 (-1.05 – 1.88)	.35/.42				0.10 (-0.02 – 0.22)	.09/.23			
Adm. BE	-0.13 (-0.52 – 0.25)	.48/.03		*(17)*		-0.12 (-15.10–14.85)	.93/.01		*(3)*		-0.14 (-0.49 – 0.21)	.39/.06		*(14)*	
Adm. TCC	0.05 (-0.71 – 0.82)	.89/.001				7.43 (-10.2 – 25.1)	.21/.62				0.06 (-0.55 – 0.67)	.83/.004			
TCC-AUC$_{3-6}$	-0.03 (-0.29 – 0.23)	.82/.004				0.22 (-2.55 – 3.00)	.76/.06				-0.07 (-0.28 – 0.14)	.48/.04			
Daily TCC (9)	-0.31 (-1.55 – 0.92)	.60/.02				7.20 (1.68 – 12.71)	.03/.94				-0.64 (-1.55 – 0.26)	.15/.17			
Total model			NF					NF					NF		

[a] Major head injury was defined as maximum Abbreviated Injury Scale (AIS) severity code ≥3 in Injury Severity Score (ISS) region Head or neck.

[b] *n* is given where group size is less than *n* given in heading.

Abbreviations: V.I. = Variable Importance. M : F = Male : Female. MOI = Mechanism of injury. B : P = Blunt : Penetrating. NISS = New Injury Severity Score. Adm. = Admission. BE = Base Excess. SOFA score = Sequential Organ Failure Assessment score. NF = Not feasible. *p* values represent two-tailed probability.
