# Supplementary material for: Increased complement activation 3 to 6 h after trauma is a predictor of prolonged mechanical ventilation and multiple organ dysfunction syndrome: a prospective observational study
Source: Mol Med. 2021 Apr 8;27:35. doi: 10.1186/s10020-021-00286-3 (PMC8028580; doi:10.1186/s10020-021-00286-3)
Supplement: Supplementary file 9 — Additional file 9. Figure S4. Assessment of SOFA scores. [file 10020_2021_286_MOEM9_ESM.pdf]

A

Sequential Organ Failure Assessment score						
	Cardiovascular	Respiratory	Renal	Central nervous system	Coagulation	Liver
Points	MAP or vasopressors [mmHg or µg/kg/h]	PaO2/FiO2-ratio [kPa]	Creatinine or urine output [µM or mL/24h]	Glasgow Coma Scale [0–15]	Platelet count [x10³ /µL]	Bilirubin [µM]
0	MAP ≥ 70	> 53	< 110	15	> 150	< 20
1	MAP < 70	40– 53	110 – 170	13 – 14	101 – 150	20 – 32
2	DA < 5	26.6 – 39.9	171 – 299	10 – 12	51 – 100	33 – 101
3	DA ≥ 5 or NA/A < 0.1	13.3 – 26.5	300 – 440 or < 500 mL/24h	6 – 9	21 – 50	102 – 204
4	NA/A ≥ 0.1	< 13.3	> 440 or < 200 mL/24h	< 6	≤ 20	> 204

B

SpO_2 to PaO_2 conversion table	
SpO_2 (%)	PaO_2 (kPa)
100	13.5
99	13
98	12.5
97	12
96	11.5
95	11
94	10.5
93	10
92	9.5
91	9
90	8.5
89	8
88	7.5
87	7
86	6.5
85	6
84	5.5
83	5
82	4.5
81	4
80	3.5

C

FiO_2 conversion table	
O_2 by nasal catheter (L/min)	FiO_2
1	0.24
2	0.27
3	0.30
4	0.33
5	0.36
6	0.39

Supplemental Figure 4. Sequential Organ Failure Assessment (SOFA) scoring
A. Information on assessment of SOFA organ scores, according to Vincent JL, Moreno R, Takala J et al.: The SOFA (Sepsis-related Organ Failure Assessment) score to describe organ dysfunction/failure. On behalf of the Working Group on Sepsis-Related Problems of the European Society of Intensive Care Medicine. Intensive Care Medicine 1996; 22:707–710. **B.** Table used for conversion of measured peripheral saturation (SpO2) to arterial oxygen partial pressure (PaO2) when measured PaO2 did not exist. **C.** Table used for conversion of oxygen received on a nasal catheter to FiO2. From https://ek.helse-bergen.no/docs/pub/DOK50708.pdf, accessed 11 September 2020.
